# 4-Cyano­pyridinium nitrate

**DOI:** 10.1107/S1600536812020697

**Published:** 2012-05-12

**Authors:** Wen-Ni Zheng

**Affiliations:** a College of Chemistry and Chemical Engineering, Southeast University, Nanjing 210096, People’s Republic of China

## Abstract

The title compound, C_6_H_5_N_2_^+^·NO_3_^−^, is a proton-transfer compound between 4-cyano­pyridine and nitric acid. In the asymmetric unit, the components are linked by a strong N—H⋯O hydrogen bond. In the crystal, mol­ecules are linked into a *C*(6) chain along [010] by C—H⋯O inter­actions.

## Related literature

For the structures and ferroelectric properties of related compounds, see: Fu *et al.* (2011*a* ▶,*b* ▶,*c* ▶); Dai & Chen (2011 ▶); Xu *et al.* (2011 ▶); Zheng (2011 ▶). For graph-set motif see: Bernstein *et al.* (1995 ▶).
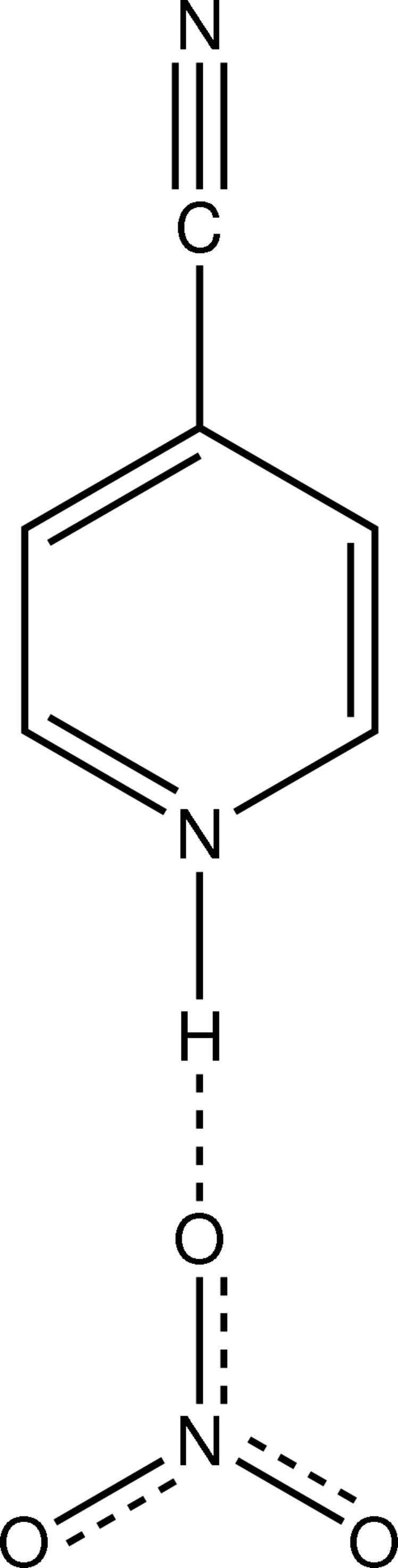


## Experimental

### 

#### Crystal data

C_6_H_5_N_2_^+^·NO_3_^−^
*M* *_r_* = 167.13
Monoclinic, 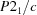

*a* = 6.3663 (2) Å
*b* = 13.2868 (9) Å
*c* = 9.1019 (2) Å
β = 103.755 (1)°
*V* = 747.83 (6) Å^3^
*Z* = 4
Mo *K*α radiation
μ = 0.12 mm^−1^
*T* = 298 K
0.10 × 0.05 × 0.05 mm

#### Data collection

Rigaku Mercury2 diffractometer
Absorption correction: multi-scan (*CrystalClear*; Rigaku, 2005 ▶) *T*_min_ = 0.910, *T*_max_ = 1.000
5151 measured reflections
1694 independent reflections
1349 reflections with *I* > 2σ(*I*)
*R*_int_ = 0.031

#### Refinement

*R*[*F* ^2^ > 2σ(*F* ^2^)] = 0.050
*wR*(*F* ^2^) = 0.146
*S* = 1.14
1694 reflections
109 parameters
1 restraint
H-atom parameters constrained
Δρ_max_ = 0.23 e Å^−3^
Δρ_min_ = −0.19 e Å^−3^

### 

Data collection: *CrystalClear* (Rigaku, 2005 ▶); cell refinement: *CrystalClear*; data reduction: *CrystalClear*; program(s) used to solve structure: *SHELXS97* (Sheldrick, 2008 ▶); program(s) used to refine structure: *SHELXL97* (Sheldrick, 2008 ▶); molecular graphics: *OLEX2* (Dolomanov *et al.*, 2009 ▶); software used to prepare material for publication: *SHELXTL* (Sheldrick, 2008 ▶).

## Supplementary Material

Crystal structure: contains datablock(s) I, global. DOI: 10.1107/S1600536812020697/bx2407sup1.cif

Structure factors: contains datablock(s) I. DOI: 10.1107/S1600536812020697/bx2407Isup2.hkl

Supplementary material file. DOI: 10.1107/S1600536812020697/bx2407Isup3.cml

Additional supplementary materials:  crystallographic information; 3D view; checkCIF report

## Figures and Tables

**Table 1:** Hydrogen-bond geometry (Å, °)

*D*—H⋯*A*	*D*—H	H⋯*A*	*D*⋯*A*	*D*—H⋯*A*
N1—H1⋯O3	0.90	1.75	2.6481 (18)	176
C4—H4*A*⋯O3^i^	0.93	2.29	3.220 (2)	179

Symmetry code: (i) 
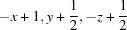
.

**Fig. 1.**
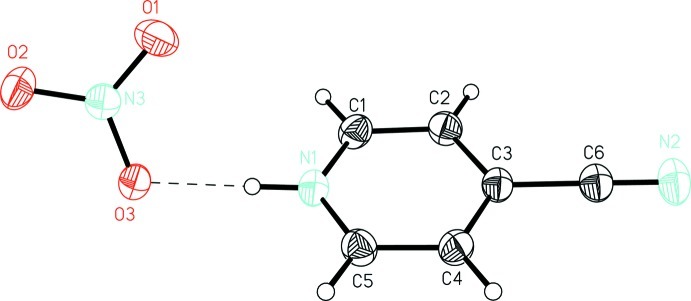
A view of the asymmetric unit with the atomic numbering scheme. The displacement ellipsoids were drawn at the 30% probability level.

**Fig. 2.**
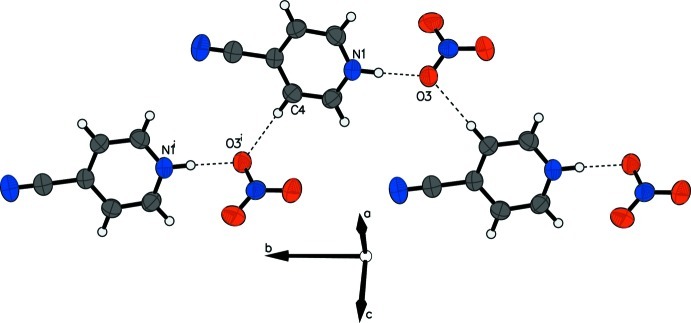
The crystal packing of the title compound viewed along the a axis showing the N—H···O and C—H···O interactions (dotted line) in the title compound. : symmetry code (i): -x+1, y+1/2, -z+1/2.

## supplementary crystallographic information

### Comment

Simple organic salts containing strong intermolecular H-bonds have attracted an attention as materials which display ferroelectric-paraelectric phase transitions (Fu *et al.*, 2011*a*, 2011*b*, 2011*c*). With the purpose of obtaining phase transition crystals of organic salts, various organic molecules have been studied and a series of new materials have been prepared (Dai & Chen 2011; Xu, *et al.* 2011; Zheng 2011). We report here the crystal structure of the title compound. The title compound (C_6_H_5_N_2_)^+^.NO_3_^- ^is a proton-transfer compound between 4-cyanopyridine and nitric acid. In the asymmetric unit the components are linked by one strong N—H···O hydrogen bond interaction, Fig 1. In the crystal the molecules are linked into C(6) chain by simple C—H···O interactions along [010] (Bernstein, *et al.*, 1995), (Fig. 2, Table1).

### Experimental

The HNO_3_ (5 mL), isonicotinonitrile (20 mmol) and ethanol (50 mL) were added into a 100mL flask. The mixture was stirred at 60^o^C for 2 h, and then the precipitate was filtrated out. Colourless crystals suitable for X-ray diffraction were obtained by slow evaporation of the solution.

### Refinement

All the H atoms attached to C atoms were situated into the idealized positions and treated as riding with C–H = 0.93 Å (aromatic) with *U_iso_*(H)=1.2*U_eq_*(C). The positional parameters of the H atom (N) was refined freely and in the last stage of the refinement, it was restrained with the N—H = 0.90Å, with *U_iso_*(H)=1.2*U_eq_*(N).

### Crystal data

**Table:**

C_6_H_5_N_2_^+^·NO_3_^−^	*F*(000) = 344
*M**_r_* = 167.13	*D*_x_ = 1.484 Mg m^−^^3^
Monoclinic, *P*2_1_/*c*	Mo *K*α radiation, λ = 0.71073 Å
Hall symbol: -P 2ybc	Cell parameters from 1694 reflections
*a* = 6.3663 (2) Å	θ = 2.8–27.5°
*b* = 13.2868 (9) Å	µ = 0.12 mm^−^^1^
*c* = 9.1019 (2) Å	*T* = 298 K
β = 103.755 (1)°	Block, colourless
*V* = 747.83 (6) Å^3^	0.10 × 0.05 × 0.05 mm
*Z* = 4	

### Data collection

**Table:**

Rigaku Mercury2 diffractometer	1694 independent reflections
Radiation source: fine-focus sealed tube	1349 reflections with *I* > 2σ(*I*)
Graphite monochromator	*R*_int_ = 0.031
Detector resolution: 13.6612 pixels mm^-1^	θ_max_ = 27.5°, θ_min_ = 2.8°
CCD profile fitting scans	*h* = −8→7
Absorption correction: multi-scan (*CrystalClear*; Rigaku, 2005)	*k* = −17→15
*T*_min_ = 0.910, *T*_max_ = 1.000	*l* = −11→11
5151 measured reflections	

### Refinement

**Table:**

Refinement on *F*^2^	Primary atom site location: structure-invariant direct methods
Least-squares matrix: full	Secondary atom site location: difference Fourier map
*R*[*F*^2^ > 2σ(*F*^2^)] = 0.050	Hydrogen site location: inferred from neighbouring sites
*wR*(*F*^2^) = 0.146	H-atom parameters constrained
*S* = 1.14	*w* = 1/[σ^2^(*F*_o_^2^) + (0.0719*P*)^2^ + 0.0927*P*] where *P* = (*F*_o_^2^ + 2*F*_c_^2^)/3
1694 reflections	(Δ/σ)_max_ < 0.001
109 parameters	Δρ_max_ = 0.23 e Å^−^^3^
1 restraint	Δρ_min_ = −0.19 e Å^−^^3^

### Special details

**Table:**

Geometry. All esds (except the esd in the dihedral angle between two l.s. planes) are estimated using the full covariance matrix. The cell esds are taken into account individually in the estimation of esds in distances, angles and torsion angles; correlations between esds in cell parameters are only used when they are defined by crystal symmetry. An approximate (isotropic) treatment of cell esds is used for estimating esds involving l.s. planes.
Refinement. Refinement of F^2^ against ALL reflections. The weighted R-factor wR and goodness of fit S are based on F^2^, conventional R-factors R are based on F, with F set to zero for negative F^2^. The threshold expression of F^2^ > 2sigma(F^2^) is used only for calculating R-factors(gt) etc. and is not relevant to the choice of reflections for refinement. R-factors based on F^2^ are statistically about twice as large as those based on F, and R- factors based on ALL data will be even larger.

### Fractional atomic coordinates and isotropic or equivalent isotropic displacement parameters (Å^2^)

**Table:**

	*x*	*y*	*z*	*U*_iso_*/*U*_eq_	
O3	0.7551 (2)	0.46541 (8)	0.20815 (14)	0.0477 (4)	
N3	0.7428 (2)	0.42233 (11)	0.08092 (16)	0.0447 (4)	
N1	0.7453 (2)	0.66359 (10)	0.17652 (16)	0.0440 (4)	
H1	0.7413	0.5962	0.1860	0.053*	
C3	0.7627 (3)	0.86509 (12)	0.13720 (18)	0.0400 (4)	
N2	0.7637 (3)	1.05763 (13)	0.0946 (2)	0.0648 (5)	
C4	0.6008 (3)	0.82380 (12)	0.1963 (2)	0.0456 (4)	
H4A	0.4974	0.8648	0.2230	0.055*	
O2	0.7350 (2)	0.32899 (10)	0.07536 (16)	0.0631 (4)	
C2	0.9185 (3)	0.80337 (13)	0.0992 (2)	0.0455 (4)	
H2A	1.0287	0.8304	0.0603	0.055*	
C5	0.5955 (3)	0.72140 (13)	0.2149 (2)	0.0466 (4)	
H5A	0.4877	0.6924	0.2543	0.056*	
O1	0.7392 (3)	0.47362 (11)	−0.03265 (17)	0.0684 (5)	
C1	0.9049 (3)	0.70149 (13)	0.1208 (2)	0.0474 (4)	
H1A	1.0073	0.6587	0.0966	0.057*	
C6	0.7672 (3)	0.97304 (14)	0.1142 (2)	0.0484 (5)	

### Atomic displacement parameters (Å^2^)

**Table:**

	*U*^11^	*U*^22^	*U*^33^	*U*^12^	*U*^13^	*U*^23^
O3	0.0566 (8)	0.0384 (6)	0.0478 (7)	−0.0022 (5)	0.0118 (6)	−0.0026 (5)
N3	0.0376 (8)	0.0469 (8)	0.0511 (9)	0.0017 (6)	0.0136 (6)	−0.0006 (6)
N1	0.0483 (8)	0.0339 (7)	0.0491 (8)	−0.0012 (6)	0.0102 (6)	0.0000 (6)
C3	0.0417 (9)	0.0359 (8)	0.0399 (8)	−0.0008 (6)	0.0047 (6)	0.0005 (6)
N2	0.0756 (13)	0.0408 (9)	0.0743 (12)	−0.0017 (8)	0.0107 (10)	0.0084 (7)
C4	0.0448 (9)	0.0431 (9)	0.0513 (10)	0.0059 (7)	0.0165 (8)	0.0003 (7)
O2	0.0760 (10)	0.0423 (8)	0.0727 (10)	−0.0014 (6)	0.0211 (8)	−0.0115 (6)
C2	0.0419 (9)	0.0464 (9)	0.0506 (10)	−0.0030 (7)	0.0157 (7)	0.0009 (7)
C5	0.0448 (10)	0.0457 (10)	0.0513 (10)	−0.0031 (8)	0.0153 (8)	0.0032 (7)
O1	0.0877 (11)	0.0705 (10)	0.0542 (8)	0.0080 (8)	0.0313 (7)	0.0124 (7)
C1	0.0466 (10)	0.0447 (9)	0.0527 (10)	0.0046 (8)	0.0155 (8)	−0.0042 (7)
C6	0.0504 (11)	0.0429 (10)	0.0495 (9)	−0.0015 (7)	0.0071 (8)	0.0018 (7)

### Geometric parameters (Å, º)

**Table:**

O3—N3	1.2774 (18)	C3—C6	1.451 (2)
N3—O1	1.234 (2)	N2—C6	1.137 (2)
N3—O2	1.2418 (19)	C4—C5	1.373 (2)
N1—C5	1.334 (2)	C4—H4A	0.9300
N1—C1	1.337 (2)	C2—C1	1.374 (2)
N1—H1	0.9005	C2—H2A	0.9300
C3—C4	1.385 (2)	C5—H5A	0.9300
C3—C2	1.392 (2)	C1—H1A	0.9300
			
O1—N3—O2	121.67 (16)	C3—C4—H4A	120.6
O1—N3—O3	119.80 (15)	C1—C2—C3	118.17 (15)
O2—N3—O3	118.53 (14)	C1—C2—H2A	120.9
C5—N1—C1	122.52 (15)	C3—C2—H2A	120.9
C5—N1—H1	120.6	N1—C5—C4	119.88 (16)
C1—N1—H1	116.9	N1—C5—H5A	120.1
C4—C3—C2	120.22 (15)	C4—C5—H5A	120.1
C4—C3—C6	119.33 (15)	N1—C1—C2	120.31 (16)
C2—C3—C6	120.44 (15)	N1—C1—H1A	119.8
C5—C4—C3	118.89 (15)	C2—C1—H1A	119.8
C5—C4—H4A	120.6	N2—C6—C3	177.8 (2)

### Hydrogen-bond geometry (Å, º)

**Table:**

*D*—H···*A*	*D*—H	H···*A*	*D*···*A*	*D*—H···*A*
N1—H1···O3	0.90	1.75	2.6481 (18)	176
C4—H4*A*···O3^i^	0.93	2.29	3.220 (2)	179

Symmetry code: (i) −*x*+1, *y*+1/2, −*z*+1/2.
